# Chromosome X-Wide Association Study Identifies Loci for Fasting Insulin and Height and Evidence for Incomplete Dosage Compensation

**DOI:** 10.1371/journal.pgen.1004127

**Published:** 2014-02-06

**Authors:** Taru Tukiainen, Matti Pirinen, Antti-Pekka Sarin, Claes Ladenvall, Johannes Kettunen, Terho Lehtimäki, Marja-Liisa Lokki, Markus Perola, Juha Sinisalo, Efthymia Vlachopoulou, Johan G. Eriksson, Leif Groop, Antti Jula, Marjo-Riitta Järvelin, Olli T. Raitakari, Veikko Salomaa, Samuli Ripatti

**Affiliations:** 1 Institute for Molecular Medicine Finland (FIMM), University of Helsinki, Helsinki, Finland; 2 Analytic and Translational Genetics Unit, Massachusetts General Hospital, Boston, Massachusetts, United States of America; 3 Program in Medical and Population Genetics, Broad Institute of Harvard and MIT, Cambridge, Massachusetts, United States of America; 4 Unit of Public Health Genomics, National Institute for Health and Welfare, Helsinki, Finland; 5 Department of Clinical Sciences, Diabetes and Endocrinology, Lund University and Lund University Diabetes Centre, CRC at Skåne University Hospital, Malmö, Sweden; 6 Department of Clinical Chemistry, Fimlab Laboratories, University of Tampere School of Medicine, Tampere, Finland; 7 Transplantation Laboratory, Haartman Institute, University of Helsinki, Helsinki, Finland; 8 Department of Chronic Disease Prevention, National Institute for Health and Welfare, Helsinki, Finland; 9 Estonian Genome Center, University of Tartu, Tartu, Estonia; 10 Division of Cardiology, Department of Medicine, Helsinki University Central Hospital, Helsinki, Finland; 11 Department of General Practice and Primary Healthcare, University of Helsinki, Helsinki, Finland; 12 Unit of General Practice, Helsinki University Central Hospital, Helsinki, Finland; 13 Folkhälsan Research Center, Helsinki, Finland; 14 Vaasa Central Hospital, Vaasa, Finland; 15 Population Studies Unit, Department of Chronic Disease Prevention, National Institute for Health and Welfare, Turku, Finland; 16 Department of Epidemiology and Biostatistics, MRC Health Protection Agency (HPA) Centre for Environment and Health, School of Public Health, Imperial College London, London, United Kingdom; 17 Institute of Health Sciences, University of Oulu, Oulu, Finland; 18 Biocenter Oulu, University of Oulu, Oulu, Finland; 19 Unit of Primary Care, Oulu University Hospital, Oulu, Finland; 20 Department of Children and Young People and Families, National Institute for Health and Welfare, Oulu, Finland; 21 Department of Clinical Physiology and Nuclear Medicine, University of Turku and Turku University Hospital, Turku, Finland; 22 Research Centre of Applied and Preventive Cardiovascular Medicine, University of Turku, Turku, Finland; 23 Wellcome Trust Sanger Institute, Hinxton, Cambridge, United Kingdom; 24 Hjelt Institute, University of Helsinki, Helsinki, Finland; The University of Queensland, Australia

## Abstract

The X chromosome (chrX) represents one potential source for the “missing heritability” for complex phenotypes, which thus far has remained underanalyzed in genome-wide association studies (GWAS). Here we demonstrate the benefits of including chrX in GWAS by assessing the contribution of 404,862 chrX SNPs to levels of twelve commonly studied cardiometabolic and anthropometric traits in 19,697 Finnish and Swedish individuals with replication data on 5,032 additional Finns. By using a linear mixed model, we estimate that on average 2.6% of the additive genetic variance in these twelve traits is attributable to chrX, this being in proportion to the number of SNPs in the chromosome. In a chrX-wide association analysis, we identify three novel loci: two for height (rs182838724 near *FGF16/ATRX/MAGT1*, joint P-value = 2.71×10^−9^, and rs1751138 near *ITM2A*, P-value = 3.03×10^−10^) and one for fasting insulin (rs139163435 in Xq23, P-value = 5.18×10^−9^). Further, we find that effect sizes for variants near *ITM2A*, a gene implicated in cartilage development, show evidence for a lack of dosage compensation. This observation is further supported by a sex-difference in *ITM2A* expression in whole blood (P-value = 0.00251), and is also in agreement with a previous report showing *ITM2A* escapes from X chromosome inactivation (XCI) in the majority of women. Hence, our results show one of the first links between phenotypic variation in a population sample and an XCI-escaping locus and pinpoint *ITM2A* as a potential contributor to the sexual dimorphism in height. In conclusion, our study provides a clear motivation for including chrX in large-scale genetic studies of complex diseases and traits.

## Author Summary

The X chromosome (chrX) analyses have often been neglected in large-scale genome-wide association studies. Given that chrX contains a considerable proportion of DNA, we wanted to examine how the variation in the chromosome contributes to commonly studied phenotypes. To this end, we studied the associations of over 400,000 chrX variants with twelve complex phenotypes, such as height, in almost 25,000 Northern European individuals. Demonstrating the value of assessing chrX associations, we found that as a whole the variation in the chromosome influences the levels of many of these phenotypes and further identified three new genomic regions where the variants associate with height or fasting insulin levels. In one of these three associated regions, the region near *ITM2A*, we observed that there is a sex difference in the genetic effects on height in a manner consistent with a lack of dosage compensation in this locus. Further supporting this observation, *ITM2A* has been shown to be among those chrX genes where the X chromosome inactivation is incomplete. Identifying phenotype associations in regions like this where chrX allele dosages are not balanced between men and women can be particularly valuable in helping us to understand why some characteristics differ between sexes.

## Introduction

Genome-wide association studies (GWAS) have discovered a wealth of loci associated with complex phenotypes with almost 5,800 significant associations for more than 500 different phenotypes reported in the NHGRI GWAS catalog [1] (accessed August 13, 2013). These GWAS discoveries are, however, concentrated on the autosomes leaving the sex chromosomes, especially the relatively large X chromosome (chrX), underrepresented; while chrX contains approximately 5% of genomic DNA, hence being comparable in size to chromosome 7, and encodes for more than 1,500 genes, only around 20 unique significantly associated X-chromosomal loci in total are recorded in the catalog. For instance, there are hundreds of known autosomal loci for height, BMI and blood lipids, but only one significant height locus has been identified in chrX, and this in individuals of African ancestry, and no X-chromosomal associations for these other highly polygenic phenotypes have been reported. Nevertheless, almost 1% of genetic variance in height and BMI has been shown to be accountable to chrX SNPs [2], demonstrating that common genetic variation in chrX contributes to complex phenotypes.

A likely explanation for the dearth of association findings in chrX is that the chromosome is often neglected in GWAS: Wise et al. recently surveyed all published GWAS from 2010 and 2011 and found that only 33% of these studies had included chrX analyses [3]. While some association studies have opted for including chrX, such as recent genetic screens on sex-hormone binding globulin levels [4] and Grave's disease [5], removal of non-autosomal data appears to be a common practice in GWAS [6], [7]. There are many potential reasons for the exclusion of chrX in GWAS, as outlined by Wise et al [3], a major contributor being that the analysis pipeline applied for autosomes is not directly applicable to chrX analyses.

While women carry two copies of chrX, men are hemizygous for the chromosome. The allele dosages between the sexes are balanced by random X chromosome inactivation (XCI) that silences one of the two chromosomes in women, hence requiring the allele coding for chrX markers to be adjusted accordingly for the analyses. However, XCI does not evenly cover the whole of chrX, but approximately 15% of the loci in the chromosome completely escape from XCI and in further 10% of the sites the silenced chrX is variably active in women, although the expression from the inactivated copy of chrX is often lower than from the active chrX [8]. The incomplete XCI adds another layer of analytical challenges, yet at the same time it also makes chrX particularly interesting to study, as the regions of incomplete dosage compensation are among the genomic contributors to the differences between gene dosages in men and women. As such, these loci could partly explain phenotypic sexual dimorphisms and additionally contribute to the phenotypic characteristics observed in chrX aneuploidies.

Given the underutilization of chrX data in previous studies and hence the potential for novel biological discoveries, we aimed at surveying the contribution of the chromosome to complex traits. To this end, we expanded the marker set for chrX by imputing the non-pseudoautosomal region of chrX in almost 25,000 Finnish and Swedish individuals from seven discovery and one replication cohort (Table 1) by utilizing the recently released comprehensive reference panel from the 1000 Genomes Project [9]. We focused our chrX-wide screen on twelve quantitative anthropometric and cardiometabolic phenotypes for which hundreds of autosomal, but no X-chromosomal, loci have been identified in GWAS of individuals of European ancestry, namely height, body-mass-index (BMI), waist-hip-ratio (WHR), systolic and diastolic blood pressure (SPB and DBP), C-reactive protein, fasting insulin and glucose, total, LDL and HDL cholesterol (TC, LDL-C and HDL-C) and triglycerides (TG). By using a linear mixed model, we show that the variation in chrX influences the levels of many of these complex phenotypes and in an association analysis identify and replicate three new associated X-chromosomal loci, one for fasting insulin and two for height, hence demonstrating the value of assessing chrX associations. Further, we find strong evidence for a lack of dosage compensation in one of the two associated height loci by applying a meta-analysis that allows for sex heterogeneity in effects and by a formal statistical model comparison between the different dosage compensation models given the observed data.

**Table 1:** A summary of the characteristics of the discovery and replication cohorts.

Cohort	Full name	Sex	N	Age (years)	Height (cm)	BMI (kg/m^2^)
**Discovery**						
NFBC	Northern Finland Birth Cohort 1966	*Males*	2388	31.0±0.0	178.2±6.4	25.2±3.6
		*Females*	2644	31.0±0.0	164.8±6.2	24.2±4.7
COROGENE	The COROGENE Study	*Males*	2441	60.4±12.8	176.0±6.7	27.4±4.2
		*Females*	1502	62.8±13.4	161.6±6.6	26.9±5.2
DGI	Diabetes Genetics Initiative	*Males*	1534	61.0±10.6	174.9±6.3	27.5±3.7
		*Females*	1608	62.5±10.7	161.7±6.1	27.8±4.7
GENMETS	Health2000 GenMets Study	*Males*	1000	49.2±10.4	176.4±6.6	27.3±3.9
		*Females*	1040	52.1±11.4	162.7±6.5	27.2±5.0
YFS	The Cardiovascular Risk in Young Finns Study	*Males*	917	37.6±5.1	179.7±6.7	26.8±4.3
		*Females*	1111	37.6±5.0	166.0±6.0	25.3±5.0
PredictCVD	Case control sample from the FINRISK surveys	*Males*	1180	51.5±13.0	174.5±6.8	27.6±4.3
		*Females*	686	52.2±13.4	161.5±6.5	27.3±5.3
HBCS	Helsinki Birth Cohort Study	*Males*	696	61.4±2.8	176.9±5.8	27.5±4.3
		*Females*	950	61.5±3.0	163.2±5.8	27.7±5.1
**Replication**						
FINRISK	Subset from the FINRISK 1997 and 2002 surveys	*Males*	2287	46.8±13.4	175.8±7.0	26.7±4.1
		*Females*	2745	45.1±12.4	162.6±6.2	26.2±5.1

N: maximum number of individuals with phenotype and genotype data available; BMI: Body-mass-index; Age, height and BMI are given as mean ± standard deviation.

## Results

### Genetic variance in chrX contributes to the levels of many anthropometric and metabolic phenotypes

We first estimated the proportion of variance in each of the twelve phenotypes accountable jointly to the common and low-frequency (minor allele frequency (MAF) >1%) SNPs in chrX using a linear mixed model [2], [10] in the study samples for which the individual-level genotype data were available (Materials and Methods). ChrX variants were estimated to contribute to the levels of many of these phenotypes (Table 2): under the model of equal variance in males and females (see Materials and Methods for discussion about the models), more than 0.5% of the variance in height, SBP, HDL-C, fasting glucose and insulin appear to be due to chrX variation, hence motivating the search for associated variants in chrX. The highest estimate for X-linked variance (1.4%, P-value = 2.00×10^−6^) was observed for height, a highly heritable and polygenic phenotype. For the other phenotypes the statistical significance of the estimates was not overwhelming, a result of the available sample size and lower trait heritability, but also the estimates for SBP, HDL-C and fasting insulin were significantly different (P-value<0.05) from zero. In these four phenotypes, which showed non-zero X-linked variance, on average 4.0% (range 2.4%–6.0%) of the estimated whole-genome genetic variance was attributable to chrX while the corresponding value over all twelve traits was 2.6%.

**Table 2:** Estimates of the explained variances in the twelve quantitative phenotypes attributable to chromosome X SNPs and autosomal SNPs separately using equal variance (EV) model.

Phenotype	N	h_X_ (%)	se_X_ (%)	P-value	h_aut_ (%)	se_aut_ (%)
Height	14408	1.41	0.41	2.00E-06	52.35	2.41
SBP	9990	1.07	0.52	0.005	16.63	2.98
Fasting glucose	9151	0.84	0.57	0.06	11.15	3.09
HDL-C	11139	0.73	0.42	0.01	30.21	2.85
Fasting insulin	9616	0.68	0.47	0.04	12.66	2.98
TG	11140	0.43	0.4	0.1	19.88	2.72
CRP	9697	0.42	0.47	0.2	11.16	2.89
WHR	12334	0.22	0.34	0.2	12.75	2.36
BMI	14214	0.11	0.31	0.4	25.86	2.25
DBP	9984	0	0.42	0.5	12.5	2.89
LDL-C	11040	0	0.41	0.5	26.43	2.82
TC	11141	0	0.43	0.5	27.21	2.81

The estimates are based on an analysis of the individuals from six Finnish cohorts using the program GCTA and 217,112 common and low-frequency chrX SNPs (MAF>1%) directly genotyped or imputed with high-quality (info >0.8) and 319,445 directly genotyped autosomal SNPs (MAF>1%).

h_X_: estimate for the proportion of explained variance accountable by the SNPs in chromosome X in per cent; se_X_: standard error in per cent for the X chromosome variance estimate; P-value: P-value for the test of h_X_ = 0; h_aut_: estimate for the proportion of explained variance accountable by the SNPs in autosomes in per cent; se_aut_: standard error in per cent for the autosomal variance estimate; SBP: systolic blood pressure; HDL-C: high-density lipoprotein cholesterol; TG: total triglycerides; CRP: C-reactive protein; WHR: waist-hip-ratio; BMI: body-mass-index; DBP: diastolic blood pressure; LDL-C: low-density lipoprotein cholesterol; TC: total cholesterol.

Following the work of Yang et al. [2] we calculated the variance estimates under three different models for dosage compensation, i.e., equal variance between men and women, full dosage compensation and no dosage compensation (Table S1). The differences between the model fit were small (as measured by likelihood-ratios) and none of the models was consistently favored above the other two. This is likely due to our sample size being limited for such comparisons, but may also reflect the differences in the genetic architecture of the various loci in chrX that contribute to the variance for each phenotype.

### ChrX-wide association analysis identifies three associated loci

**Figure 1:**
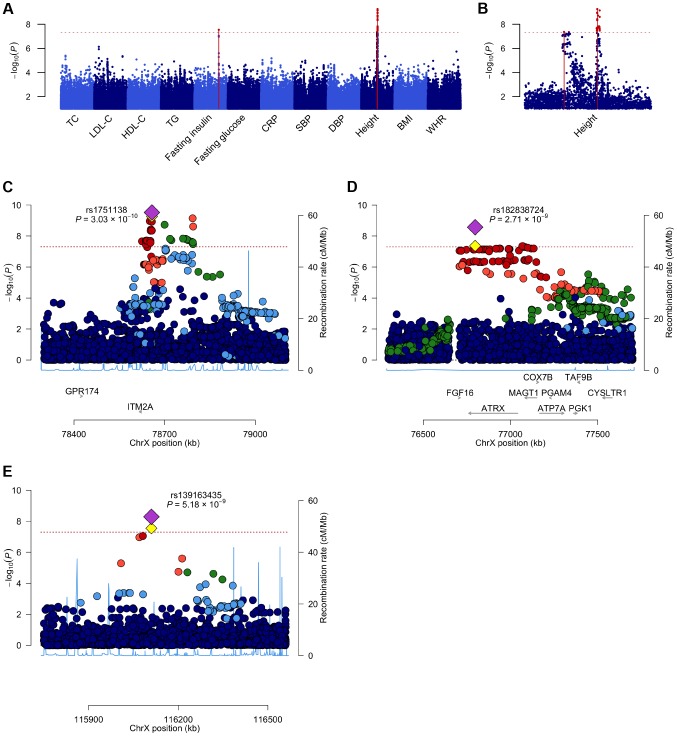
A Manhattan plot across all the twelve phenotypes and regional association plots for the three associated loci. A. A Manhattan plot showing the associations of the X chromosome SNPs to the twelve phenotypes in the discovery analysis. The associated loci (P-value<5.0×10^−8^) are highlighted with red dots and solid lines. B. A plot of the height associations in the Xq21.1 region showing two separate association signals. C–E. The association plots for height near *ITM2A* (C) and height near *ATRX* (D) and for fasting insulin in Xq23 (E), showing the association P-values in the discovery analysis. Yellow diamonds indicate the SNPs, which showed the strongest evidence of association in each of the loci, and purple diamonds and the P-values given in the plots indicate the associations of these lead SNPs in the joint analysis of discovery and replication data sets. Each circle in the plots indicates a SNP with the color of the circle (in C–E) showing the linkage disequilibrium between the SNP and the highlighted lead SNP: dark blue (r^2^<0.2), light blue (r^2^>0.2), green (r^2^>0.4), orange (r^2^>0.6) and red (r^2^>0.8), The r^2^ values were calculated using the genotype data from the COROGENE cohort, and the recombination rate, indicated by the blue lines in the background and the right hand y-axis, was estimated from the CEU HapMap data. The bottom panels show the genes (RefSeq Genes) and their positions in each locus. In all plots the dashed red line marks the threshold for genome-wide significance (P-value = 5.0×10^−8^).

In order to identify X-linked loci contributing to the phenotypic variance we assessed associations between directly genotyped and imputed chrX SNPs and the twelve phenotypes across seven discovery cohorts (N = 19,697; Table 1). As the majority of the loci in chrX are subject to XCI, we adopted an allele coding that is consistent with the full dosage compensation model, i.e. we treated hemizygous men as equivalent to homozygous women (Materials and Methods). Within each cohort the associations were first studied separately for males and females using SNPTEST [11] and the results were subsequently combined in a fixed-effects meta-analysis in GWAMA [12]. By analyzing the associations of up to 405,411 polymorphic high-quality SNPs we identified three associated loci (P-value<5×10^−8^): two for height (both in Xq21.1) and one for fasting insulin (in Xq23) (Table 3, Table S2, Figure 1). We followed-up these findings in an independent replication set and found further evidence for association in all three loci (discovery and replication combined up to N = 24,729), with all lead SNP P-values<6×10^−9^ (Table 3).

**Table 3:** The lead associations in the three significantly associated loci in the chromosome X-wide association analysis.

Locus/Candidates	SNP	Pos (chrX)	EA/OA	Data set	Sex	EAF	Beta	SE	P-value	N	P-value (sex)	P-value (het)	Variance explained (%)
Xq21.1/ITM2A	rs1751138	78657806	G/A	Discovery	Males+Females	0.643	0.057	0.009	5.54E-10	19566	1.99E-10	1.33E-02	-
					Males	0.641	0.045	0.011	3.32E-05	10093	-	-	0.19%
					Females	0.644	0.084	0.016	1.67E-07	9473	-	-	0.32%
				Replication	Males+Females	0.647	0.038	0.019	5.53E-02	4996	2.53E-04	3.32E-04	-
					Males	0.647	−0.013	0.024	5.94E-01	2259	-	-	0.01%
					Females	0.647	0.128	0.032	5.51E-05	2737	-	-	0.75%
				Joint	Males+Females	0.643	0.054	0.008	3.03E-10	24562	3.26E-12	2.85E-04	-
					Males	0.642	0.036	0.010	3.39E-04	12352	-	-	0.12%
					Females	0.645	0.093	0.014	2.56E-10	12210	-	-	0.39%
Xq21.1/FGF16, ATRX, MAGT1	rs182838724	76797439	T/A	Discovery	Males+Females	0.299	0.053	0.009	4.31E-08	19562	1.31E-07	1.99E-01	-
					Males	0.294	0.053	0.011	4.07E-06	10092	-	-	0.23%
					Females	0.304	0.054	0.016	1.24E-03	9470	-	-	0.12%
				Replication	Males+Females	0.290	0.056	0.020	7.74E-03	4996	1.69E-02	3.03E-01	-
					Males	0.287	0.046	0.025	6.60E-02	2259	-	-	0.18%
					Females	0.293	0.071	0.033	2.88E-02	2737	-	-	0.21%
				Joint	Males+Females	0.297	0.054	0.008	2.71E-09	24558	4.44E-09	8.19E-02	-
					Males	0.293	0.052	0.010	8.95E-07	12351	-	-	0.22%
					Females	0.301	0.058	0.015	1.58E-04	12207	-	-	0.14%
Xq23	rs139163435	116110239	G/T	Discovery	Males+Females	0.071	−0.128	0.023	2.87E-08	11681	1.72E-07	5.74E-01	-
					Males	0.071	−0.137	0.028	1.45E-06	5542	-	-	0.50%
					Females	0.071	−0.110	0.039	4.95E-03	6139	-	-	0.16%
				Replication	Males+Females	0.088	−0.221	0.110	4.51E-02	370	9.95E-02	4.39E-01	-
					Males	0.091	−0.170	0.129	1.87E-01	200	-	-	0.96%
					Females	0.084	−0.363	0.214	9.00E-02	170	-	-	2.04%
				Joint	Males+Females	0.072	−0.132	0.023	5.18E-09	12051	3.46E-08	6.65E-01	-
					Males	0.072	−0.139	0.028	6.05E-07	5742	-	-	0.51%
					Females	0.071	−0.118	0.039	2.15E-03	6309	-	-	0.19%

EA: effect allele; OA: other allele; Data set: the data sets included in the meta-analysis: discovery cohorts (discovery), the replication cohort (replication), both discovery and replication cohorts (joint); Sex: the sex in which the analysis was conducted; EAF: effect allele frequency; Beta: effect size for the effect allele; SE: standard error for Beta; P-value: P-value for the association from fixed-effects meta-analysis; N: sample size in the analysis; P-value (sex): P-value for the association from sex-differentiated meta-analysis; P-value (het): P-value from the sex heterogeneity test; Variance explained: the proportion of phenotype variance the lead SNP explains in per cent, calculated from meta-analysis summary statistics (Materials and Methods).

In the more strongly associated height locus the associated SNPs (lead SNP rs1751138, joint, i.e., discovery and replication combined, P-value = 3.03×10^−10^, MAF = 0.36) are located approximately 35 kb upstream of *ITM2A* (integral membrane protein 2A), a gene implicated in early cartilage development [13], [14]. We observed that the minor A allele of rs1751138, which is associated with shorter stature, is also associated with an increased expression of *ITM2A* in whole blood (P-value = 6.23×10^−14^, N = 513; Materials and Methods), providing further evidence for *ITM2A* as a functional candidate gene for this association. The second region associated with height spans *FGF16*, *ATRX* and *MAGT1*. The lead SNP (rs182838724, joint P-value = 2.71×10^−9^, MAF = 0.30) is intronic within *ATRX*, a gene associated with the X-linked alpha thalassaemia mental retardation syndrome (ATR-X), a rare condition manifesting itself as profound developmental delay often accompanied by several other distinct characteristics including skeletal abnormalities in 90% and short stature in two thirds of the affected individuals [15]. As the two height lead SNPs map only 2 Mb apart, we confirmed that the associations are independent of each other by conditioning the association analysis on the lead SNP of the *ITM2A* locus. The conditional analysis did not attenuate the height signal in the *ATRX* region, yet here the most associated SNP (rs34979608, joint P-value = 1.52×10^−9^, r^2^ with rs182838724 = 0.91; Table S3, Figure S1) maps outside *ATRX*, 4 kb downstream of *MAGT1*, a gene encoding a magnesium transporter. Both of the height associations are present already in childhood (P-value for *ITM2A* = 1.58×10^−5^, P-value for *ATRX* = 0.00955, N = 3287, subset of two of the study cohorts, ages 8–10; Table S4), but for the *ATRX* locus the association appears weaker in children than in the same individuals in adulthood (beta in childhood = 0.059, beta in adulthood = 0.092, P-value for difference in effect sizes = 0.043; Table S4) suggesting additional influence of puberty. In the third associated locus, the dosage of the minor G allele of rs139163435 (MAF = 0.071) was robustly associated with lower levels of fasting insulin across all cohorts (joint P-value = 5.18×10^−9^). The lead SNP maps to an apparent gene desert: the gene closest to the association, *SLC6A14*, a possible candidate gene for X-linked obesity (OMIM: 300444), lies more than 500 kb away.

### Genetic effects near ITM2A suggest a lack of dosage compensation

Up to 25% of the chrX loci may not be subject to complete dosage compensation [8], but in these regions also the inactivated X chromosomes are transcriptionally fully or partially active. When adopting an allele coding that is consistent with the full dosage compensation model, as we did in the cohort-level analyses, the genetic effects in these incompletely dosage compensated loci are expected to be larger in absolute value in women than in men. The fixed-effects meta-analysis does not, however, account for potential sex heterogeneity. Therefore, we complemented the fixed-effects analysis by performing a meta-analysis that treats the male and female-specific genetic effects separately, a so-called sex-differentiated meta-analysis (Materials and Methods), in order to indicate loci showing incomplete dosage compensation and to potentially also facilitate the discovery of new associations.

Allowing for different effect sizes between males and females in the meta-analysis pinpointed no further loci, yet the *ITM2A* lead height SNP was more strongly associated in this sex-differentiated analysis (joint P-value = 3.26×10^−12^; Table 3). Pointing to lack of dosage compensation, in this locus the allelic effects were estimated to be more than twice the size in women compared to men when coding hemizygous men equal to homozygous women (standardized beta in females: 0.093, se: 0.014; beta in males: 0.035, se: 0.009; P-value for the difference in the male and female effects = 2.85×10^−4^; Table 3). For the other two new loci there was no indication of heterogeneity in the male and female effects (Table 3). Accordingly, the proportion of variance explained was approximately twice the size in men compared to women in the *ATRX* (0.22% vs. 0.14% for height) and Xq23 (0.51% vs. 0.19% for fasting insulin) loci, as expected under the model of random XCI, but not for the *ITM2A* SNP (0.12% vs. 0.39% for height) (explained variances calculated under the assumption of full dosage compensation; Table 3; Materials and Methods). The observed deviation from the full dosage compensation model in the *ITM2A* locus was not driven by differences in allele frequencies or sample sizes, as these are similar between men and women (Tables 1 and 3).

### Building up evidence for a lack of dosage compensation in the ITM2A locus

**Figure 2:**
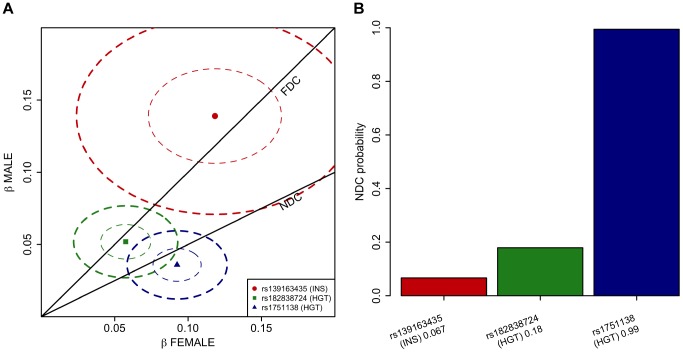
Comparison of the dosage compensation models in the three associated loci applying Bayesian framework. A. Separately estimated effect sizes of the three lead SNPs (dots labeled with rs-numbers) in females (x-axis) and males (y-axis) when female genotypes are coded {0,1,2} and male genotypes {0,2}. Ellipses show the 95% confidence regions for the estimates. The lines show the regions of the expected values of the effects under either full dosage compensation (FDC) or no dosage compensation (NDC) models. The associated traits are fasting insulin (INS) and height (HGT). B. Posterior probability of no dosage compensation (NDC) model at the three lead SNPs when the other candidate is full dosage compensation model and the two models are equally probable *a priori*. Labels under bars give the rs-number of the SNP, the associated trait (INS = fasting insulin or HGT = height) and the height of the bar.

To verify the observation of lack of dosage compensation in the *ITM2A* locus for height, we formally investigated how the models of full dosage compensation and no dosage compensation explain the data in the three associated loci pinpointed in our chrX-wide association analysis. To this end we devised a novel application of a Bayesian model-comparison framework [16] (Materials and Methods), to quantify the relative support for each of the dosage compensation models. While the Xq23 and *ATRX* associations were highly consistent with full dosage compensation there was clear evidence towards complete escape from the inactivation in the *ITM2A* locus: Assuming that both models are equally probable *a priori*, the posterior probabilities for the no dosage compensation model are 0.07 for rs13916345 in Xq23 (fasting insulin), and 0.18 for rs182838724 near *ATRX* (height), but 0.99 for rs1751138 near *ITM2A* (height) (Figure 2).

As lack of dosage compensation in the *ITM2A* locus in women should be reflected in the level of *ITM2A* expression, given the two actively transcribed X chromosomes, we evaluated the sex difference in the level of the *ITM2A* expression probe that had earlier showed a significant *cis*-effect with the lead SNP of the locus. The average level of *ITM2A* expression in whole blood was observed to be higher in women (P-value = 0.00251; Figure S2; Materials and Methods), thus providing further support for incomplete XCI in this locus.

## Discussion

Motivated by the underrepresentation of reported GWAS discoveries in chrX, we investigated the association of chrX to twelve anthropometric and cardiometabolic traits in more than 24,500 individuals using a high-resolution map of non-pseudoautosomal chrX SNPs. Our data demonstrate that SNPs in chrX are associated with many of the studied phenotypes, including the three novel loci pinpointed in our chrX-wide association analysis. Additionally, our discovery of lack of dosage compensation for height near *ITM2A* not only highlights the value of accounting for potential sex heterogeneity when assessing chrX associations, but also manifests that some of the X-linked loci may contribute to sexual dimorphisms, in this case to the height difference between men and women.

The contribution of common X-chromosomal SNPs to a few complex phenotypes have been explored previously: Yang et al. showed that between 0.57% and 0.82% of the variance in height, BMI and von Willebrand factor is explained by the SNPs in chrX [2]. In our study, we extended the estimates to further ten anthropometric and cardiometabolic phenotypes, and also use a more comprehensive set of SNPs, and similarly demonstrate that a small but non-negligible proportion, up to 1.4%, of the total variance in many of the twelve phenotypes studied appears attributable to chrX. While the variance estimates for autosomes have been observed to be proportional to the chromosome length [2], our estimates for chrX were on average 2.6% of the total genetic variance estimate, and therefore below what would be expected based on the proportion of the genomic DNA contained within chrX, i.e., approximately 5%. However, given the smaller population size and lower mutation rate, chrX is genetically less diverse than the autosomes, and indeed our estimates appear to be more in line with around 3.4% of the SNPs in the 1000 Genomes reference being X-chromosomal [9] (Figure S3). This implies that around 3% of all GWAS discoveries could be hidden in chrX and hence with the inclusion of chrX in GWAS further X-chromosomal loci for complex traits will be discovered.

As thus far chrX has only infrequently been included in GWAS of the twelve traits studied, it is unsurprising that the three loci now discovered, one for fasting insulin and two for height, represent the first ones reported in chrX for these phenotypes in European populations. Two earlier GWAS conducted in individuals of African ancestry discovered a height locus in Xp22.3 [17], [18], yet the associated lead SNP is monomorphic in Europeans and the region shows no signal in our analyses. One of the early height GWAS including mainly European samples showed a suggestive association (P-value = 3×10^−6^) [19] mapping 8.6 kb from the *ITM2A* association discovered in our analyses (r^2^ = 0.728 between the lead SNPs), however, in that study the finding failed to replicate and thus never reached the formal threshold for significance.

While further studies are required to elaborate the causative variants underlying the association with fasting insulin in Xq23, in both of the height loci there are plausible candidate genes that could be responsible for the observations. The first height association spans *FGF16*, *ATRX* and *MAGT1* in a gene rich region. Both *ATRX* and *MAGT1* have been implicated in mental retardation syndromes, and interestingly the syndrome associated with mutations in *ATRX* is often accompanied with skeletal abnormalities and short stature in the affected individuals [15]. Another candidate is *FGF16*, which is a member of fibroblast growth factor family shown to play various roles in developmental processes including morphogenesis. Mouse studies proposed a crucial role for *FGF16* in cardiac morphogenesis [20] and, recently, nonsense mutations in *FGF16* were demonstrated to associate with congenital limb malformations [21], suggesting the involvement of the gene in human skeletal development. In the second height locus, *ITM2A* is a functional candidate: The association signal maps just 35 kb upstream of the transcription start site of *ITM2A*, a gene known to be involved in cartilage development [13], [14]. Additionally, our eQTL analysis provided further evidence showing that the height-associated variants in this locus also influence the expression of *ITM2A*. Interestingly, high expression of *ITM2A* in adipose tissue stem cells has been proposed to inhibit the initiation of chondrogenesis in these cells [22]. As the allele associated with shorter stature associated with increased expression of *ITM2A*, this suggests the allelic effect to height could be mediated through the capacity to generate cartilage and bone.

Given the unique nature of chrX compared to autosomes, i.e., males being hemizygous for the chromosome and the partly incomplete XCI, which could give rise to sexual dimorphisms, we analyzed our association data also by accounting for potential heterogeneity in genetic effects between sexes. This led to the discovery of considerable sex-difference in the genetic effects for height near *ITM2A*, while for the other two associated loci there was no such evidence. A large-scale scan on 270,000 individuals for sexual dimorphisms in autosomal genetic effects for various anthropometric phenotypes identified significant sex differences only in associations for waist phenotypes and none was observed for height [23]. Additionally, the few previous height GWAS that had significant or suggestive discoveries in chrX [17]–[19] did not, to our knowledge, evaluate for potential sex heterogeneity in the chrX associations. Therefore, the sex difference in genetic effects for height in the *ITM2A* locus here is likely the first demonstration of its kind and hence warrants the investigation for potential sex heterogeneity in associations for other X-linked loci.

The difference in the genetic effects for males and females near *ITM2A* appeared fully consistent with no dosage compensation in this locus. While we cannot completely exclude the possibility that such a difference can also arise through some other type of sex-by-SNP interaction effect, we gained further confirmation for the lack of dosage compensation for height in *ITM2A* by quantifying the evidence for the two dosage compensation models in each of the associated loci using a new model comparison framework. Additionally, the gene expression data that showed higher expression of *ITM2A* in women, as expected if two X chromosomes are transcriptionally active in this locus. Similarly, expression of *ITM2A* was previously observed to be female-biased in monocytes and in the same study the *cis*-eQTL for *ITM2A* showed sex heterogeneity [24]. Furthermore, strongly speaking for the role of impartial silencing of this locus, in a comprehensive survey into XCI, *ITM2A* was found to be among the 10% of chrX genes, which variably escape from inactivation, i.e., are expressed from the inactivated copy of X (Xi) in a majority of women [8]. For comparison, the expression of *ATRX* and *MAGT1* in the other height locus (*FGF16* was not included in the survey) from Xi is fully silenced [8].

Given the converging evidence from our association study, statistical model comparison of the association data, gene expression data and literature, it seems likely that our observations of sex heterogeneity in the genetic effects for height near *ITM2A* are due to incomplete dosage compensation in this locus. Therefore, our study likely provides, to the best of our knowledge, the first link between an XCI-escaping gene and phenotypic variation in non-syndromic individuals. This discovery has several plausible implications. As increased expression of *ITM2A* links with shorter stature, the greater dosage of *ITM2A* in women compared to men may explain some of the substantial sex difference observed in height. In addition to the sex differences in the overall expression levels, incomplete dosage compensation also causes the genetic variation in the population to have different effects on the trait distribution of males and females. Assuming that rs1751138, the *ITM2A* lead SNP, is the causal variant for the observed height association, we estimate that the observed 36% frequency of the height decreasing allele accounts for 1.5% of the current difference in mean height between men and women in the Finnish population, when compared to a population that was monomorphic for the major allele at this SNP (Materials and Methods). Besides contributing to sexual dimorphisms, XCI-escaping genes are candidates for causing the abnormalities in chrX aneuploidies. Hence, our findings also highlight *ITM2A* as a potential contributor to the height phenotype often observed in individuals with an unusual number of X chromosomes [25].

To conclude, our findings illustrate the value of including the chrX in large-scale genetic studies and provide a motivation to assess the chrX associations in larger sample sizes, particularly for traits where we estimated chrX to explain part of the trait variation. We anticipate that such studies will identify further loci that contribute to the heritability of complex traits, as well as increase our understanding of their genetic architecture and underlying biology. As evidenced by our observations in the *ITM2A* locus, studying chrX association opens avenues for the discovery of links between phenotypes and loci that escape from XCI. Such associations bear potential to bring insights into the biological bases of sexually dimorphic traits such as complex diseases with different incidences between males and females.

## Materials and Methods

### Cohorts

The study included individuals from seven discovery cohorts: The Northern Finland Birth Cohort 1966 (NFBC1966), The Cardiovascular Risk in Young Finns Study (YFS), The COROGENE Study (COROGENE), Helsinki Birth Cohort Study (HBCS), the Health 2000 GenMets Study (GenMets), the Diabetes Genetics Initiative (DGI) and a prospective cardiovascular disease (CVD) case-control sample from the FINRISK collections (PredictCVD). In the replication stage a further subset of individuals from the FINRISK collections (FR) was included. A summary of the cohort characteristics is given in Table 1. All participants gave an informed consent and the data was de-identified for all analyses.

NFBC1966 is a birth cohort study of children born in 1966 in the two northernmost provinces of Finland originally designed to focus on factors affecting pre-term birth, low birth weight, and subsequent morbidity and mortality [26]. The blood sample for the DNA extraction and all phenotype data (except the childhood height measurements) used in the present study were collected at a follow-up visit when the participants were 31 years of age. The COROGENE cohort includes acute coronary syndrome patients who underwent coronary angiogram between June 2006 and March 2008 in the Helsinki University Central Hospital [27] and matched controls from the Helsinki-Vantaa region participants of FINRISK 1997, 2002, and 2007 surveys performed in Finland every five years since 1972 [28]. In the current study, the COROGENE cases were only included in the analyses of height and body-mass-index. The DGI sample consists of individuals from Sweden and Finland and was originally designed to identify loci associated with type 2 diabetes (T2D) [29]. The sample consists of patients with T2D; gender, BMI, age and geographically matched controls and discordant sibships. The current analysis was performed on 3142 individuals, including all individuals from the original analysis and individuals previously excluded after having been identified as belonging to pairs of samples identified as cryptic first degree relatives [29]. GenMets is a subset from the Health 2000 survey collected in 2000–2001 to obtain information on the most important public health problems in Finland [30]. The cohort includes metabolic syndrome cases and their matched controls aged 30 years and above. YFS is a longitudinal follow-up study of children and adolescents from all around Finland initiated in 1980 to study the cardiovascular risk from childhood to adulthood [31], and the data for the present study is from the 27-year follow-up when the participants were 30–45 years of age. The PredictCVD study comprises of incident cardiovascular disease cases and matched controls selected from the 1992, 1997, 2002 and 2007 FINRISK surveys [28]. For the analyses of the current study, the 96 individuals included and genotyped in both COROGENE and PredictCVD samples were excluded from the COROGENE data set. The FR sample used for replication includes a random, yet non-overlapping with COROGENE and PredictCVD, subset of individuals from FINRISK surveys from 1997 and 2002.

### Genotyping and imputation

The following genotyping arrays were used for genotyping the study cohorts: Illumina 370K array for NFBC1966, Illumina 610K array for COROGENE and GenMets, custom generated Illumina 670K array for YFS and HBCS, Illumina 770K array for PredictCVD, Illumina HumanCoreExome-12v1-0 for FR and Affymetrix GeneChip Human Mapping 500K Array set for DGI. The quality control procedures included removing closely related individuals (PI_HAT >0.1) by analyzing pairwise IBD relationships for all individuals in five Finnish discovery cohorts together and have been described previously in detail [29], [32]. The imputation of non-pseudoautosomal chrX variants into the study cohorts was performed on the cleaned data in each cohort using IMPUTE version 2.2.2 [33], [34]. The reference panel used in the imputation was the integrated variant set release (v3) released in March 2012 (http://mathgen.stats.ox.ac.uk/impute/data_download_1000G_phase1_integrated.html). The data were split into genomic regions of ∼5 Mb (with 250 kb (DGI) or 1 Mb (other cohorts) buffer region), using effective population size of 20000 (DGI) or 11418 and k value of 80.

### Phenotype adjustments

Within each cohort, all twelve phenotypes were adjusted for males and females separately using age (not in NFBC1966) and ten first principal components as covariates. Additionally, WHR and blood pressure measurements were adjusted for BMI. The residuals from the linear regression were inverse normal transformed to have mean 0 and standard deviation 1, and the normalized residuals were then used as phenotypes in the variance estimate and association analyses. In cohorts where the information was available, individuals on lipid-lowering medication were excluded prior the covariate adjustment for the blood lipids (TC, TG, LDL-C and HDL-C), blood-pressure medication was an exclusion criterion for systolic and diastolic blood pressure and diabetes medication for glucose and insulin. Non-fasting individuals were excluded from the analysis of glucose and insulin, and pregnant women were only included in the analysis of CRP and height. All phenotype adjustments and data normalization were done in STATA/SE 12.1 or R (version 2.11.1). The sample size for each phenotype in the discovery cohorts, i.e., the number of individuals with both genotype and phenotype information, are given in Table S5.

### Estimation of the variance explained by the X chromosome and the autosomes

We estimated how much phenotypic variance a panel of 217,112 high quality SNPs from chrX (IMPUTE2 info >0.8, MAF >0.01) explain using the linear mixed model approach implemented in GCTA (v.1.13) [10]. This analysis included six of the seven discovery cohorts (NFBC1966, COROGENE, GenMets, YFS, HBCS and PredictCVD) for which we had access to the individual genotype data. Following the work of Yang et al. [2], we applied three models for dosage compensation: full dosage compensation (FDC), no dosage compensation (NDC) and equal variance in both sexes (EV). However, none of the models was consistently favored over the other two across the traits (Table S1). In the main text we report the variance estimates from the EV model because those best capture the average genetic contribution of chrX to the population: in FDC and NDC models the variances between males and females are different and for both models the mean of the sex-specific variances is close to the single value given by the EV model in our data. Furthermore, since the traits have been normalized to have a variance of 1 within each sex, the models that assume different genetic variance between the sexes (namely FDC and NDC) should also allow different residual variances between the sexes but this has not been implemented in GCTA (v.1.13). We report the results from all three models using imputed SNPs in Table S1.

For a comparison, we also estimated the genetic variances using 9,517 directly genotyped SNPs in chrX but did not find notable differences from the results with the imputed data (Table S6). For another comparison, we estimated the variance explained by the 319,445 directly genotyped SNPs with MAF>0.01 in the autosomes (Table 2). If the variants contributing to the traits were uniformly distributed across the genome, then we would expect that chrX genetic variance is about 3% of the autosomal genetic variance, as about 3% of the genetic variation in our data is in the X chromosome. Figure S3 plots estimated autosomal genetic variance against chrX one.

All mixed model analyses excluded individuals in such a way that none of the remaining pairs of individuals had an estimated relatedness coefficient r >0.05 and the same trait values were used as with the association analyses. For comparison, the analyses were also carried out by using r = 0.025 as the relatedness cut-off (Table S7).

### Chromosome X-wide association analyses

The associations between variants and phenotypes were tested for all available genotyped or imputed SNPs in chrX separately for men and women in each cohort encoding genotypes {0,2} in men and {0,1,2} in women, i.e., assuming that one of the two X chromosomes in women is fully inactivated. In DGI the analysis was performed using a linear mixed model that can account for sample structure, as implemented in EMMAX [35]. In other cohorts the analyses were performed in SNPTEST [11] (version 2.4.0) assuming an additive genetic model and using expected genotype counts. In each cohort the results were filtered prior the meta-analysis to include only good-quality variants (SNPTEST info in women >0.4) and variants with more than three copies of the minor allele (minor allele count in women >3) resulting in between 323,564 and 383,337 variants per cohort in the discovery set.

The cohorts and genders were combined in a fixed-effects (inverse variance weighted) meta-analysis and in a sex-differentiated meta-analysis, which combines the results allowing for the allelic effects to differ between men and women and also conducts a meta-analysis separately for the results from men and from women, both meta-analysis options implemented in GWAMA [12]. Genomic control was applied for the association results of each study to account for P-value inflation arising from residual population structure in the data or other confounding factors. The meta-analysis summary was also corrected with the genomic control lambda for those phenotypes where there was indication of inflation (lambda >1.0): The genomic control lambdas for the twelve phenotypes were between 0.94–1.13 in the discovery analysis (Table S8). For each phenotype the meta-analysis results were further filtered to include only the SNPs for which the association result was available from at least two of the input files, hence association results were available for up to 405,411 SNPs. Adopting the genome-wide significance P-value threshold, P-value<5×10^−8^, three loci showed significant associations in the discovery analysis. In these associated loci the lead SNPs were imputed with high quality (imputation info >0.80) in all cohorts.

### Height associations in childhood

To investigate whether the observed associations with height near *ITM2A* and *ATRX* are present already in pre-puberty, the association of the chrX SNPs with childhood height was studied in a subset of individuals from NFBC1966 and YFS cohorts (N(males) = 1204 and N(females) = 1189 in NFBC1966; N(males) = 417 and N(females) = 478 in YFS) who had height measurements available both from pre-puberty (ages 8–10 years for NFBC1966 and 9 years for YFS) and adulthood. The height measurements were adjusted for age (in NFBC1966) and ten first principal components, the residuals were inverse normal transformed and subsequently the association of the SNP dosage with the transformed residuals was studied in SNPTEST using an additive model of association. The SNP-phenotype associations for the two height measurements were studied in both cohorts, separately for the genders, and the results were combined in a fixed-effects and a sex-differentiated meta-analysis using GWAMA applying genomic control correction. The association results for the height lead SNPs with childhood and adulthood height in these individuals are given in Table S4.

### Cis-eQTL analysis of the associated regions

A subset of the COROGENE cohort (N = 513) had both genome-wide SNP data and gene expression data from whole blood, assayed using Illumina HumanHT-12v3 Expression BeadChips, available, as described previously [36]. The associations between the three lead SNPs and all gene expression probes within 1 Mb of the SNPs was studied in SNPTEST using an additive model of association. For the one significantly associated expression probe (*ITM2A*/ILMN_2076600) we further evaluated whether the level of expression was different between the sexes by comparing the unadjusted expression in males and females using Student's t-test in R.

### Variance explained by individual loci in chromosome X

As males carry only one copy of the X chromosome, the genotype variances of the SNPs in the non-pseudoautosomal region of chrX differ between women and men. Assuming the model of complete inactivation of one the X chromosomes in women, and hence coding the X-linked alleles {0,2} in men and {0,1,2} in women, the genotype variance in men is twice that in women: *2P(1-P)* for females and *4P(1-P)* males, where *P* denotes the allele frequency of the SNP. Thus, the estimation of the variance explained from the meta-analysis summary statistics should be evaluated for the genders separately using the formulas *2P(1-P)b_F_^2^* for women and *4P(1-P)b_M_^2^* for men, where b_F_ and b_M_ denote the standardized effect sizes in women and men, respectively.

### Comparison of dosage compensation models

We applied a Bayesian framework [37] to compare full dosage compensation (FDC) and no dosage compensation (NDC) models at the top SNPs of the three associated regions. We used the estimated effect sizes b_F_ (allelic effect in females) and b_M_ (effects in males when two genotypes are coded 0 and 2) together with their standard errors to approximate the likelihood function as in [16]. The two models differ in their prior specification:

FDC: b_F_∼N(0, s^2^), b_M_∼N(0, s^2^) and cor(b_F_,b_M_) = 1

NDC: b_F_∼N(0, s^2^), b_M_∼N(0, 0.25 s^2^) and cor(b_F_,b_M_) = 1

Where s^2^, the variance of the prior effect size in females, depends on the allele frequency of the SNP and is chosen so that with 95% probability the studied SNP explains less than 1% of the variance of the trait. Intuitively, according to the FDC model b_F_ = b_M_ whereas according to the NDC model b_F_ = 2b_M_. Bayes factors between the two models can be computed using the approximate likelihood approach [16] and the posterior probabilities of the NDC model are shown in Figure 2 under the assumption that each model is equally likely *a priori*.

### Effect of lack of dosage compensation on sex difference in mean trait value

Suppose that each copy of the minor allele (‘a’ with frequency f_a_) at a particular SNP on chrX causally affects the trait value by ‘b’ both in males and in females, i.e., there is no dosage compensation. To simplify notation, we assume that the mean trait value of males with genotype ‘A’ is the same as the mean trait value in females with genotype ‘AA’, and denote this mean by ‘m’. The overall mean trait values in males, ‘m_M_’, and in females, ‘m_F_’, are

m_M_ = (1−f_a_)•m + f_a_•(m+b) = m + f_a_•b, and

m_F_ = f_AA_•m + f_Aa_•(m+b) + f_aa_•(m+2b) = m + (f_Aa_+2f_aa_)•b = m + 2f_a_•b

The difference in the sex-specific means is

m_M_ − m_F_ = m + f_a_•b − (m + 2f_a_•b) = − f_a_•b

So the effect of allele ‘a’ is either to increase (if b<0) or decrease (if b>0) the male-female difference in the mean trait value by |f_a_•b|, compared to the situation where only allele ‘A’ was present in the population.

Application to rs1751138, the lead SNP of the association near *ITM2A*:

b = −0.555 cm (se = 0.0734 cm). This is a fixed-effects estimate of the allelic effects in quantile normalized height in females −0.092559 (0.013934) and males −0.071802 (0.019274), multiplied by an estimate of the standard deviation of height in Finland, 6.5 cm.

Thus by introducing the minor allele (A) with frequency f_a_ = 0.36 in the population, the male-female difference in mean height increases by 0.36*0.555 cm = 0.20 cm. As the mean difference in height between the sexes in Finland is about 13.7 cm, the variation at this SNP accounts for 0.20 cm/13.7 cm = 0.0146≈1.5% of that difference.

## Supporting Information

Figure S1 Regional association plots of the associations in the two height loci after conditioning the association analysis on rs1751138, the lead associated SNP in the *ITM2A* height locus. A: The region near *FGF16*, *ATRX* and *MAGT1*. The lead associated SNP in this analysis, rs34979608, is highlighted with diamonds, the yellow diamond indicating the association in the discovery analysis and the purple diamond and text the association in the joint analysis of discovery and replication cohorts. The yellow × indicates the association of the SNP that was most associated with height in this region in the discovery meta-analysis, i.e., before the conditional analysis (P-values 3.02×10^−7^ and 2.80×10^−8^ in the conditional analysis using the discovery cohorts and both discovery and replication data, respectively). B: The region near *ITM2A*. No association with height remains in this region in the conditional analysis. Each circle in the plots indicates a SNP and the color of the circle shows the linkage disequilibrium, r^2^, of the SNP to the lead associated SNP in each region, rs34979608 in A and rs1751138 in B: dark blue (r^2^<0.2), light blue (r^2^>0.2), green (r^2^>0.4), orange (r^2^>0.6) and red (r^2^>0.8). The correlation structure between the SNPs was calculated from Finnish data using the genotypes from the COROGENE cohort. The light blue line in the background and the right hand y-axis show the recombination rate in the region as calculated from HapMap CEU data.
(PDF)

Figure S2 Boxplots of *ITM2A* expression in men and women. The levels of whole blood *ITM2A* expression visualized using boxplots separately for men (blue) and women (orange) in the individuals of COROGENE cohort (N = 513) for whom expression data was available. The mean expression level is higher in women (P-value = 0.00251) providing support for incomplete dosage compensation between men and women in the *ITM2A* locus.
(PDF)

Figure S3 Comparison of the phenotypic variances attributable to chromosome X variants (y-axis) and autosomal variants (x-axis). The dashed line is y = 0.03x and shows the region of the expected values of the points under the assumption that the genetic effects are small and uniformly distributed across the genome and that the X chromosome contains about 3% of all genetic variation. The dotted lines show the standard errors of the estimates. The traits are TC: total cholesterol; LDL-C: low-density lipoprotein cholesterol; HDL-C: high-density lipoprotein cholesterol; TG: total triglycerides; CRP: C-reactive protein; BMI: body-mass-index; WHR: waist-hip-ratio; SBP: systolic blood pressure; DBP: diastolic blood pressure; HGT: height; GLK: fasting glucose; INS: fasting insulin.
(PNG)

Table S1 Estimates of the phenotypic variance attributable to 217,112 imputed and genotyped chrX SNPs using three different dosage compensation models.
(XLS)

Table S2 Association results in each discovery cohort for the lead SNPs in the three associated loci (rs1751138 near *ITM2A* and rs182838724 near *FGF16*, *ATRX* and *MAGT1* for height and rs139163435 in Xq23 for fasting insulin).
(XLS)

Table S3 Lead associations in the height locus near *FGF16*, *ATRX* and *MAGT1* after conditioning the association analysis on the lead SNP of the *ITM2A* locus, rs1751138.
(XLS)

Table S4 Associations of the most associated SNPs in the two height loci with height in childhood (age 8–10) and adulthood in a subset of the study subjects from two cohorts (NFBC1966 and YFS) for whom childhood height measurements were available.
(XLS)

Table S5 Sample sizes for the twelve phenotypes in the discovery cohorts.
(XLS)

Table S6 Estimates of the phenotypic variance attributable to 9,517 directly genotyped chrX SNPs using three different dosage compensation models.
(XLS)

Table S7 Estimates of the phenotypic variance attributable to 217,112 imputed and genotyped chrX SNPs using three different dosage compensation models using a threshold of 0.025 to remove related individuals.
(XLS)

Table S8 Genomic control lambdas for the twelve phenotypes in the X chromosome-wide meta-analyses of the discovery cohorts. In cases where lambda >1, the meta-analysis results were corrected using the lambda statistic.
(XLS)
