# Supplementary figures and images for: Chromosome X-Wide Association Study Identifies Loci for Fasting Insulin and Height and Evidence for Incomplete Dosage Compensation
Source: PLoS Genet. 2014 Feb 6;10(2):e1004127. doi: 10.1371/journal.pgen.1004127 (PMC3916240; doi:10.1371/journal.pgen.1004127)

**A**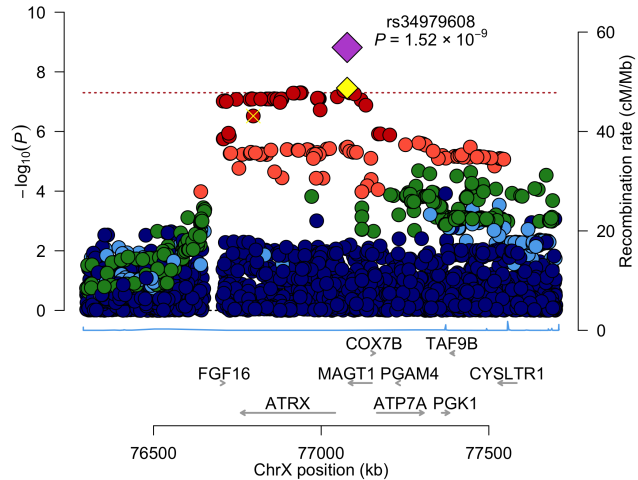**B**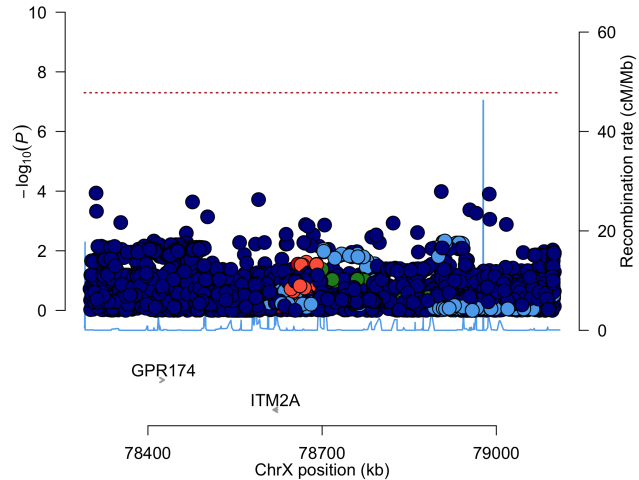

Supplement: Figure S1 — Regional association plots of the associations in the two height loci after conditioning the association analysis on rs1751138, the lead associated SNP in the ITM2A height locus. A: The region near FGF16, ATRX and MAGT1. The lead associated SNP in this analysis, rs34979608, is highlighted with diamonds, the yellow diamond indicating the association in the discovery analysis and the purple diamond and text the association in the joint analysis of discovery and replication cohorts. The yellow × indicates the association of the SNP that was most associated with height in this region in the discovery meta-analysis, i.e., before the conditional analysis (P-values 3.02×10^−7^ and 2.80×10^−8^ in the conditional analysis using the discovery cohorts and both discovery and replication data, respectively). B: The region near ITM2A. No association with height remains in this region in the conditional analysis. Each circle in the plots indicates a SNP and the color of the circle shows the linkage disequilibrium, r2, of the SNP to the lead associated SNP in each region, rs34979608 in A and rs1751138 in B: dark blue (r2<0.2), light blue (r2>0.2), green (r2>0.4), orange (r2>0.6) and red (r2>0.8). The correlation structure between the SNPs was calculated from Finnish data using the genotypes from the COROGENE cohort. The light blue line in the background and the right hand y-axis show the recombination rate in the region as calculated from HapMap CEU data. (PDF) [file pgen.1004127.s001.pdf]

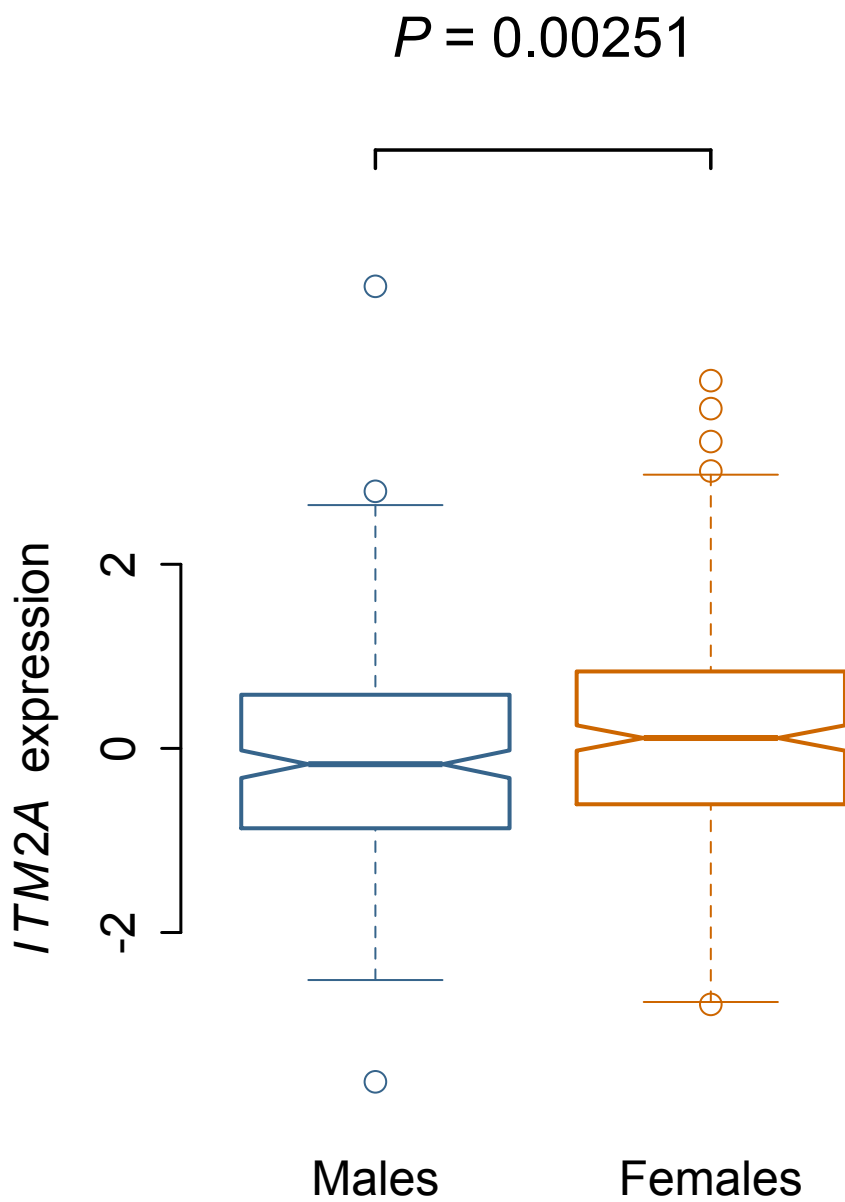

Supplement: Figure S2 — Boxplots of ITM2A expression in men and women. The levels of whole blood ITM2A expression visualized using boxplots separately for men (blue) and women (orange) in the individuals of COROGENE cohort (N = 513) for whom expression data was available. The mean expression level is higher in women (P-value = 0.00251) providing support for incomplete dosage compensation between men and women in the ITM2A locus. (PDF) [file pgen.1004127.s002.pdf]

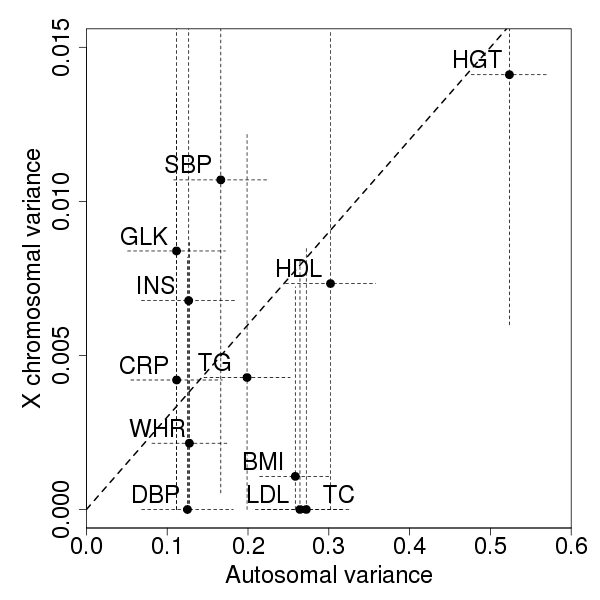

Supplement: Figure S3 — Comparison of the phenotypic variances attributable to chromosome X variants (y-axis) and autosomal variants (x-axis). The dashed line is y = 0.03x and shows the region of the expected values of the points under the assumption that the genetic effects are small and uniformly distributed across the genome and that the X chromosome contains about 3% of all genetic variation. The dotted lines show the standard errors of the estimates. The traits are TC: total cholesterol; LDL-C: low-density lipoprotein cholesterol; HDL-C: high-density lipoprotein cholesterol; TG: total triglycerides; CRP: C-reactive protein; BMI: body-mass-index; WHR: waist-hip-ratio; SBP: systolic blood pressure; DBP: diastolic blood pressure; HGT: height; GLK: fasting glucose; INS: fasting insulin. (PNG) [file pgen.1004127.s003.png]
